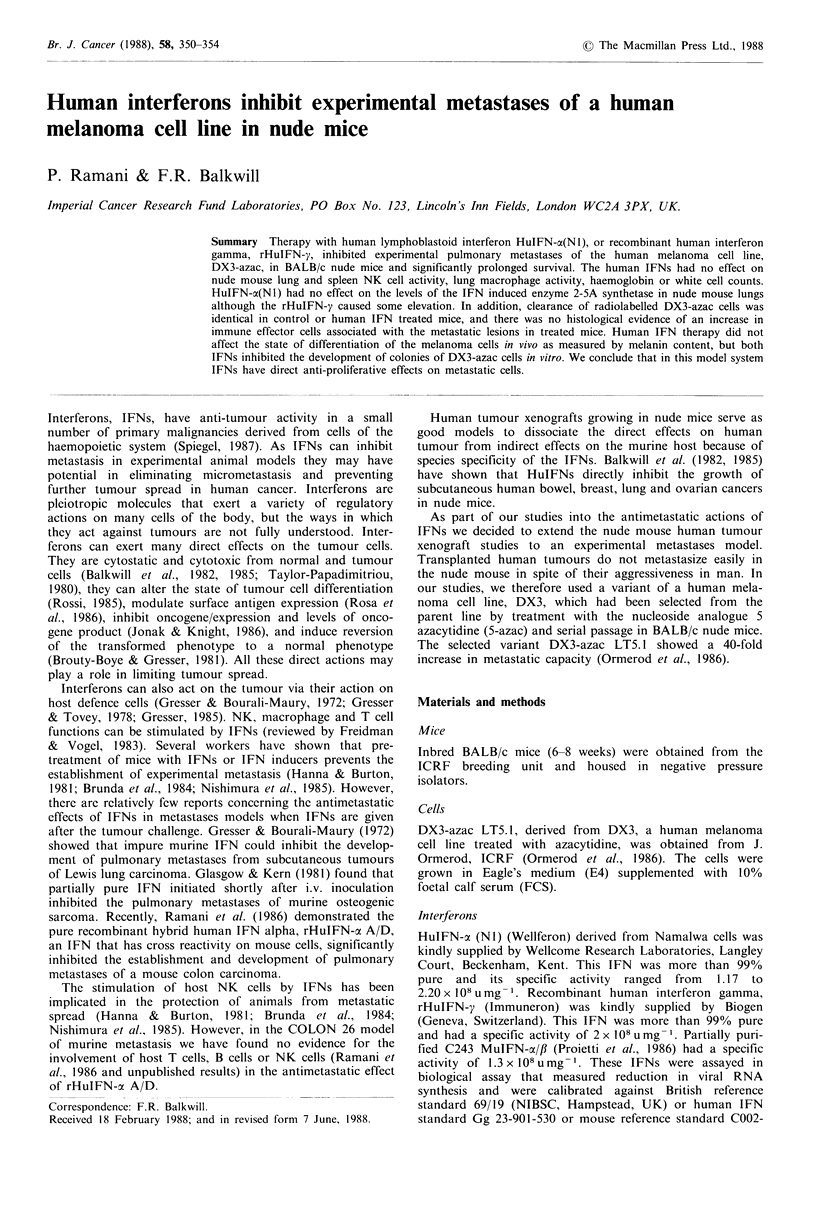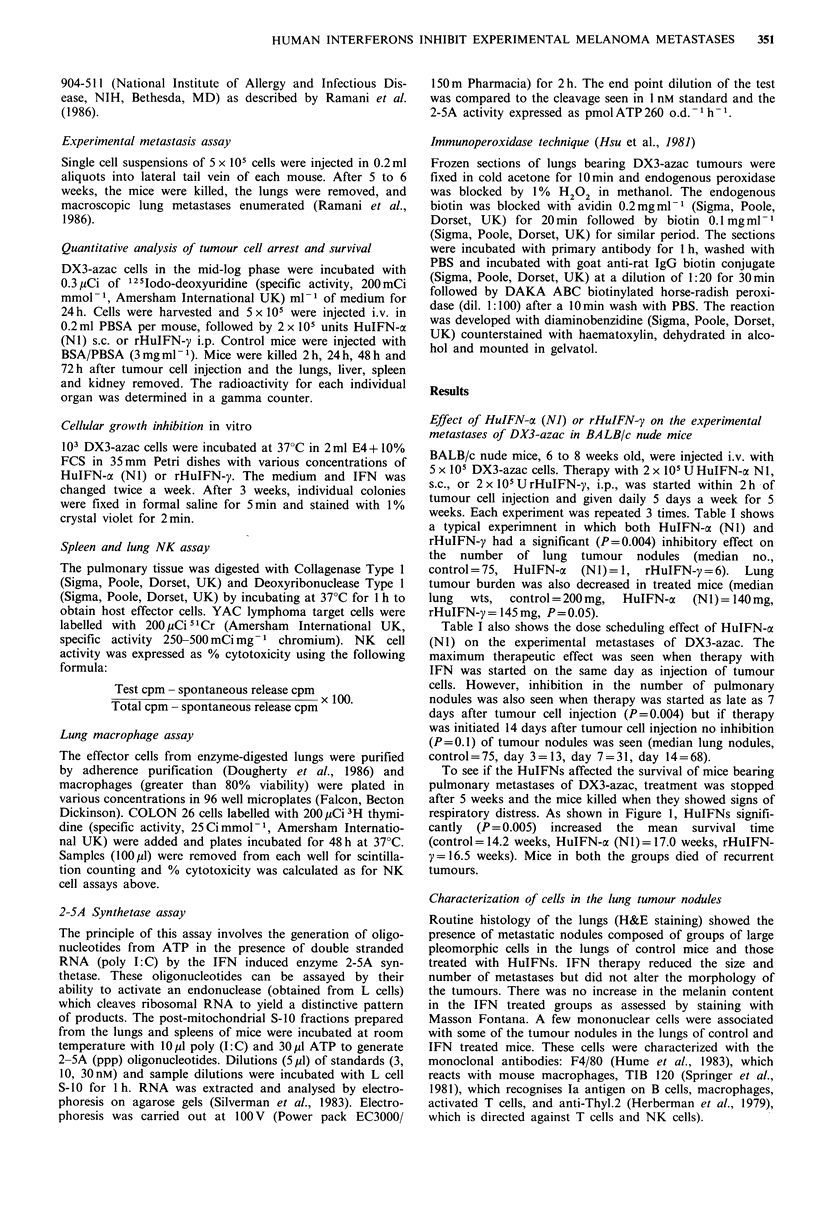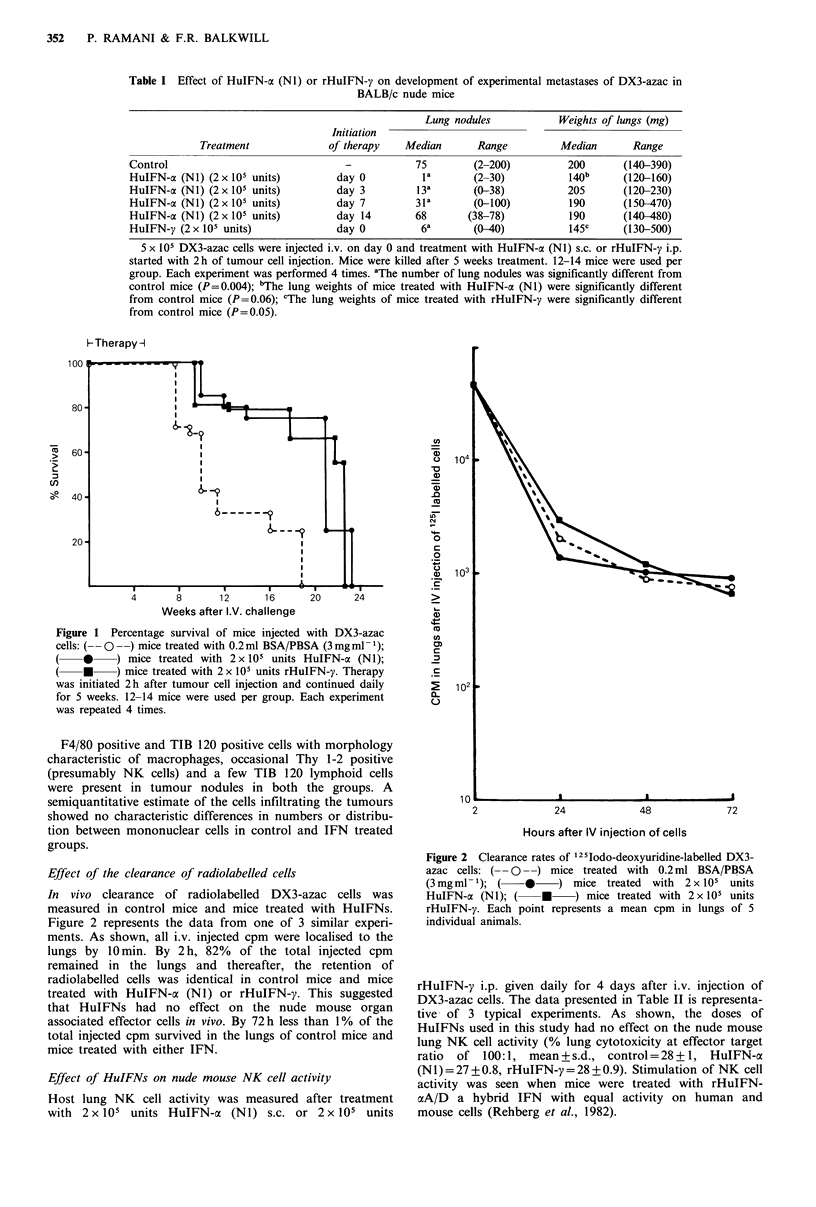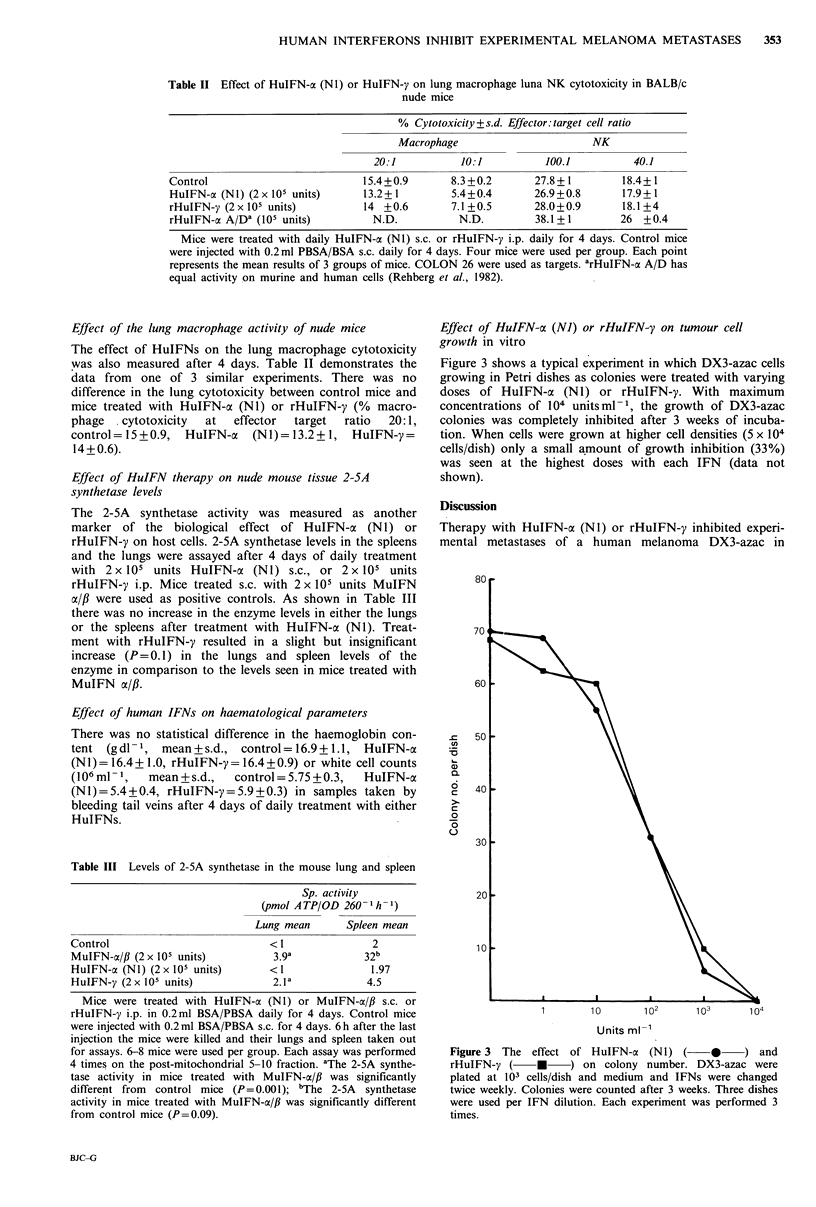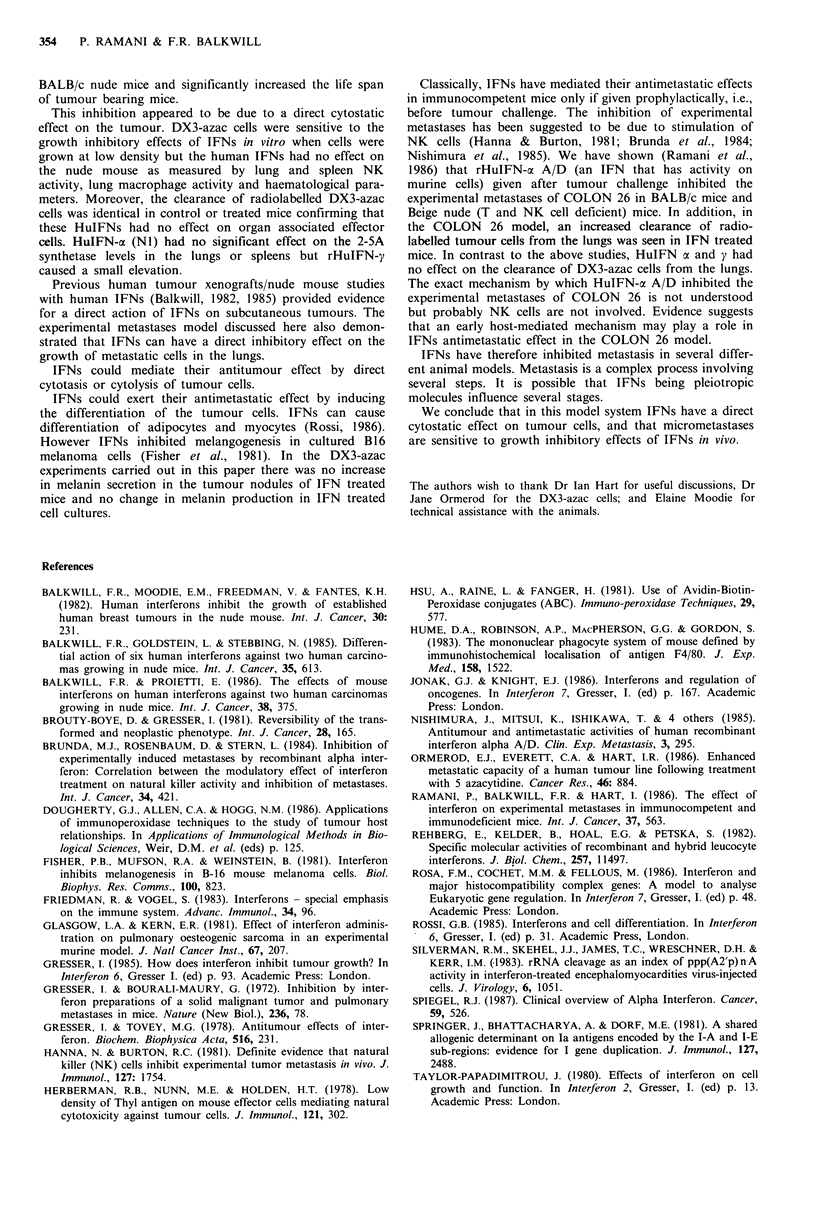# Human interferons inhibit experimental metastases of a human melanoma cell line in nude mice.

**DOI:** 10.1038/bjc.1988.217

**Published:** 1988-09

**Authors:** P. Ramani, F. R. Balkwill

**Affiliations:** Imperial Cancer Research Fund Laboratories, London, UK.

## Abstract

Therapy with human lymphoblastoid interferon HuIFN-alpha(N1), or recombinant human interferon gamma, rHuIFN-gamma, inhibited experimental pulmonary metastases of the human melanoma cell line, DX3-azac, in BALB/c nude mice and significantly prolonged survival. The human IFNs had no effect on nude mouse lung and spleen NK cell activity, lung macrophage activity, haemoglobin or white cell counts. HuIFN-alpha(N1) had no effect on the levels of the IFN induced enzyme 2-5A synthetase in nude mouse lungs although the rHuIFN-gamma caused some elevation. In addition, clearance of radiolabelled DX3-azac cells was identical in control or human IFN treated mice, and there was no histological evidence of an increase in immune effector cells associated with the metastatic lesions in treated mice. Human IFN therapy did not affect the state of differentiation of the melanoma cells in vivo as measured by melanin content, but both IFNs inhibited the development of colonies of DX3-azac cells in vitro. We conclude that in this model system IFNs have direct anti-proliferative effects on metastatic cells.


*Br. J. Cancer* (1988), **58**, 350–354 © The Macmillan Press Ltd., 1988

### Human interferons inhibit experimental metastases of a human melanoma cell line in nude mice

P. Ramani & F.R. Balkwill

*Imperial Cancer Research Fund Laboratories, PO Box No. 123, Lincoln's Inn Fields, London WC2A 3PX, UK.*

**Summary** Therapy with human lymphoblastoid interferon HuIFN-α(N1), or recombinant human interferon gamma, rHuIFN-γ, inhibited experimental pulmonary metastases of the human melanoma cell line, DX3-azac, in BALB/c nude mice and significantly prolonged survival. The human IFNs had no effect on nude mouse lung and spleen NK cell activity, lung macrophage activity, haemoglobin or white cell counts. HuIFN-α(N1) had no effect on the levels of the IFN induced enzyme 2-5A synthetase in nude mouse lungs although the rHuIFN-γ caused some elevation. In addition, clearance of radiolabelled DX3-azac cells was identical in control or human IFN treated mice, and there was no histological evidence of an increase in immune effector cells associated with the metastatic lesions in treated mice. Human IFN therapy did not affect the state of differentiation of the melanoma cells *in vivo* as measured by melanin content, but both IFNs inhibited the development of colonies of DX3-azac cells *in vitro*. We conclude that in this model system IFNs have direct anti-proliferative effects on metastatic cells.

Interferons, IFNs, have anti-tumour activity in a small number of primary malignancies derived from cells of the haemopoietic system (Spiegel, 1987). As IFNs can inhibit metastasis in experimental animal models they may have potential in eliminating micrometastasis and preventing further tumour spread in human cancer. Interferons are pleiotropic molecules that exert a variety of regulatory actions on many cells of the body, but the ways in which they act against tumours are not fully understood. Interferons can exert many direct effects on the tumour cells. They are cytostatic and cytotoxic from normal and tumour cells (Balkwill *et al.*, 1982, 1985; Taylor-Papadimitriou, 1980), they can alter the state of tumour cell differentiation (Rossi, 1985), modulate surface antigen expression (Rosa *et al.*, 1986), inhibit oncogene/expression and levels of oncogene product (Jonak & Knight, 1986), and induce reversion of the transformed phenotype to a normal phenotype (Brouty-Boye & Gresser, 1981). All these direct actions may play a role in limiting tumour spread.

Interferons can also act on the tumour via their action on host defence cells (Gresser & Bourali-Maury, 1972; Gresser & Tovey, 1978; Gresser, 1985). NK, macrophage and T cell functions can be stimulated by IFNs (reviewed by Freidman & Vogel, 1983). Several workers have shown that pretreatment of mice with IFNs or IFN inducers prevents the establishment of experimental metastasis (Hanna & Burton, 1981; Brunda *et al.*, 1984; Nishimura *et al.*, 1985). However, there are relatively few reports concerning the antimetastatic effects of IFNs in metastases models when IFNs are given after the tumour challenge. Gresser & Bourali-Maury (1972) showed that impure murine IFN could inhibit the development of pulmonary metastases from subcutaneous tumours of Lewis lung carcinoma. Glasgow & Kern (1981) found that partially pure IFN initiated shortly after i.v. inoculation inhibited the pulmonary metastases of murine osteogenic sarcoma. Recently, Ramani *et al.* (1986) demonstrated the pure recombinant hybrid human IFN alpha, rHuIFN-α A/D, an IFN that has cross reactivity on mouse cells, significantly inhibited the establishment and development of pulmonary metastases of a mouse colon carcinoma.

The stimulation of host NK cells by IFNs has been implicated in the protection of animals from metastatic spread (Hanna & Burton, 1981; Brunda *et al.*, 1984; Nishimura *et al.*, 1985). However, in the COLON 26 model of murine metastasis we have found no evidence for the involvement of host T cells, B cells or NK cells (Ramani *et al.*, 1986 and unpublished results) in the antimetastatic effect of rHuIFN-α A/D.

Correspondence: F.R. Balkwill.
Received 18 February 1988; and in revised form 7 June, 1988.

Human tumour xenografts growing in nude mice serve as good models to dissociate the direct effects on human tumour from indirect effects on the murine host because of species specificity of the IFNs. Balkwill *et al.* (1982, 1985) have shown that HuIFNs directly inhibit the growth of subcutaneous human bowel, breast, lung and ovarian cancers in nude mice.

As part of our studies into the antimetastatic actions of IFNs we decided to extend the nude mouse human tumour xenograft studies to an experimental metastases model. Transplanted human tumours do not metastasize easily in the nude mouse in spite of their aggressiveness in man. In our studies, we therefore used a variant of a human melanoma cell line, DX3, which had been selected from the parent line by treatment with the nucleoside analogue 5 azacytidine (5-azac) and serial passage in BALB/c nude mice. The selected variant DX3-azac LT5.1 showed a 40-fold increase in metastatic capacity (Ormerod *et al.*, 1986).

### Materials and methods

### *Mice*

Inbred BALB/c mice (6–8 weeks) were obtained from the ICRF breeding unit and housed in negative pressure isolators.

### *Cells*

DX3-azac LT5.1, derived from DX3, a human melanoma cell line treated with azacytidine, was obtained from J. Ormerod, ICRF (Ormerod *et al.*, 1986). The cells were grown in Eagle's medium (E4) supplemented with 10% foetal calf serum (FCS).

### *Interferons*

HuIFN-α (N1) (Wellferon) derived from Namalwa cells was kindly supplied by Wellcome Research Laboratories, Langley Court, Beckenham, Kent. This IFN was more than 99% pure and its specific activity ranged from 1.17 to $2.20 \times 10^8\,\mathrm{u\,mg^{-1}}$. Recombinant human interferon gamma, rHuIFN-γ (Immuneron) was kindly supplied by Biogen (Geneva, Switzerland). This IFN was more than 99% pure and had a specific activity of $2 \times 10^8\,\mathrm{u\,mg^{-1}}$. Partially purified C243 MuIFN-α/β (Proietti *et al.*, 1986) had a specific activity of $1.3 \times 10^8\,\mathrm{u\,mg^{-1}}$. These IFNs were assayed in biological assay that measured reduction in viral RNA synthesis and were calibrated against British reference standard 69/19 (NIBSC, Hampstead, UK) or human IFN standard Gg 23-901-530 or mouse reference standard C002-

904-511 (National Institute of Allergy and Infectious Disease, NIH, Bethesda, MD) as described by Ramani *et al.* (1986).

*Experimental metastasis assay*

Single cell suspensions of $5 \times 10^5$ cells were injected in 0.2 ml aliquots into lateral tail vein of each mouse. After 5 to 6 weeks, the mice were killed, the lungs were removed, and macroscopic lung metastases enumerated (Ramani *et al.*, 1986).

*Quantitative analysis of tumour cell arrest and survival*

DX3-azac cells in the mid-log phase were incubated with 0.3 $\mu$Ci of $^{125}$Iodo-deoxyuridine (specific activity, 200 mCi mmol$^{-1}$, Amersham International UK) ml$^{-1}$ of medium for 24 h. Cells were harvested and $5 \times 10^5$ were injected i.v. in 0.2 ml PBSA per mouse, followed by $2 \times 10^5$ units HuIFN-$\alpha$ (N1) s.c. or rHuIFN-$\gamma$ i.p. Control mice were injected with BSA/PBSA (3 mg ml$^{-1}$). Mice were killed 2 h, 24 h, 48 h and 72 h after tumour cell injection and the lungs, liver, spleen and kidney removed. The radioactivity for each individual organ was determined in a gamma counter.

*Cellular growth inhibition* in vitro

$10^3$ DX3-azac cells were incubated at 37°C in 2 ml E4 + 10% FCS in 35 mm Petri dishes with various concentrations of HuIFN-$\alpha$ (N1) or rHuIFN-$\gamma$. The medium and IFN was changed twice a week. After 3 weeks, individual colonies were fixed in formal saline for 5 min and stained with 1% crystal violet for 2 min.

*Spleen and lung NK assay*

The pulmonary tissue was digested with Collagenase Type 1 (Sigma, Poole, Dorset, UK) and Deoxyribonuclease Type 1 (Sigma, Poole, Dorset, UK) by incubating at 37°C for 1 h to obtain host effector cells. YAC lymphoma target cells were labelled with 200 $\mu$Ci $^{51}$Cr (Amersham International UK, specific activity 250–500 mCi mg$^{-1}$ chromium). NK cell activity was expressed as % cytotoxicity using the following formula:

$$\frac{\text{Test cpm} - \text{spontaneous release cpm}}{\text{Total cpm} - \text{spontaneous release cpm}} \times 100.$$

*Lung macrophage assay*

The effector cells from enzyme-digested lungs were purified by adherence purification (Dougherty *et al.*, 1986) and macrophages (greater than 80% viability) were plated in various concentrations in 96 well microplates (Falcon, Becton Dickinson). COLON 26 cells labelled with 200 $\mu$Ci $^3$H thymidine (specific activity, 25 Ci mmol$^{-1}$, Amersham International UK) were added and plates incubated for 48 h at 37°C. Samples (100 $\mu$l) were removed from each well for scintillation counting and % cytotoxicity was calculated as for NK cell assays above.

*2-5A Synthetase assay*

The principle of this assay involves the generation of oligonucleotides from ATP in the presence of double stranded RNA (poly I:C) by the IFN induced enzyme 2-5A synthetase. These oligonucleotides can be assayed by their ability to activate an endonuclease (obtained from L cells) which cleaves ribosomal RNA to yield a distinctive pattern of products. The post-mitochondrial S-10 fractions prepared from the lungs and spleens of mice were incubated at room temperature with 10 $\mu$l poly (I:C) and 30 $\mu$l ATP to generate 2–5A (ppp) oligonucleotides. Dilutions (5 $\mu$l) of standards (3, 10, 30 nM) and sample dilutions were incubated with L cell S-10 for 1 h. RNA was extracted and analysed by electrophoresis on agarose gels (Silverman *et al.*, 1983). Electrophoresis was carried out at 100 V (Power pack EC3000/150 m Pharmacia) for 2 h. The end point dilution of the test was compared to the cleavage seen in 1 nM standard and the 2-5A activity expressed as pmol ATP 260 o.d.$^{-1}$ h$^{-1}$.

*Immunoperoxidase technique (Hsu* et al., *1981)*

Frozen sections of lungs bearing DX3-azac tumours were fixed in cold acetone for 10 min and endogenous peroxidase was blocked by 1% $H_2O_2$ in methanol. The endogenous biotin was blocked with avidin 0.2 mg ml$^{-1}$ (Sigma, Poole, Dorset, UK) for 20 min followed by biotin 0.1 mg ml$^{-1}$ (Sigma, Poole, Dorset, UK) for similar period. The sections were incubated with primary antibody for 1 h, washed with PBS and incubated with goat anti-rat IgG biotin conjugate (Sigma, Poole, Dorset, UK) at a dilution of 1:20 for 30 min followed by DAKA ABC biotinylated horse-radish peroxidase (dil. 1:100) after a 10 min wash with PBS. The reaction was developed with diaminobenzidine (Sigma, Poole, Dorset, UK) counterstained with haematoxylin, dehydrated in alcohol and mounted in gelvatol.

### Results

*Effect of HuIFN-$\alpha$ (N1) or rHuIFN-$\gamma$ on the experimental metastases of DX3-azac in BALB/c nude mice*

BALB/c nude mice, 6 to 8 weeks old, were injected i.v. with $5 \times 10^5$ DX3-azac cells. Therapy with $2 \times 10^5$ U HuIFN-$\alpha$ N1, s.c., or $2 \times 10^5$ U rHuIFN-$\gamma$, i.p., was started within 2 h of tumour cell injection and given daily 5 days a week for 5 weeks. Each experiment was repeated 3 times. Table I shows a typical experimnent in which both HuIFN-$\alpha$ (N1) and rHuIFN-$\gamma$ had a significant ($P = 0.004$) inhibitory effect on the number of lung tumour nodules (median no., control = 75, HuIFN-$\alpha$ (N1) = 1, rHuIFN-$\gamma$ = 6). Lung tumour burden was also decreased in treated mice (median lung wts, control = 200 mg, HuIFN-$\alpha$ (N1) = 140 mg, rHuIFN-$\gamma$ = 145 mg, $P = 0.05$).

Table I also shows the dose scheduling effect of HuIFN-$\alpha$ (N1) on the experimental metastases of DX3-azac. The maximum therapeutic effect was seen when therapy with IFN was started on the same day as injection of tumour cells. However, inhibition in the number of pulmonary nodules was also seen when therapy was started as late as 7 days after tumour cell injection ($P = 0.004$) but if therapy was initiated 14 days after tumour cell injection no inhibition ($P = 0.1$) of tumour nodules was seen (median lung nodules, control = 75, day 3 = 13, day 7 = 31, day 14 = 68).

To see if the HuIFNs affected the survival of mice bearing pulmonary metastases of DX3-azac, treatment was stopped after 5 weeks and the mice killed when they showed signs of respiratory distress. As shown in Figure 1, HuIFNs significantly ($P = 0.005$) increased the mean survival time (control = 14.2 weeks, HuIFN-$\alpha$ (N1) = 17.0 weeks, rHuIFN-$\gamma$ = 16.5 weeks). Mice in both the groups died of recurrent tumours.

*Characterization of cells in the lung tumour nodules*

Routine histology of the lungs (H&E staining) showed the presence of metastatic nodules composed of groups of large pleomorphic cells in the lungs of control mice and those treated with HuIFNs. IFN therapy reduced the size and number of metastases but did not alter the morphology of the tumours. There was no increase in the melanin content in the IFN treated groups as assessed by staining with Masson Fontana. A few mononuclear cells were associated with some of the tumour nodules in the lungs of control and IFN treated mice. These cells were characterized with the monoclonal antibodies: F4/80 (Hume *et al.*, 1983), which reacts with mouse macrophages, TIB 120 (Springer *et al.*, 1981), which recognises Ia antigen on B cells, macrophages, activated T cells, and anti-Thyl.2 (Herberman *et al.*, 1979), which is directed against T cells and NK cells).

**Table I** Effect of HuIFN-α (N1) or rHuIFN-γ on development of experimental metastases of DX3-azac in BALB/c nude mice

| Treatment | Initiation of therapy | Lung nodules | | Weights of lungs (mg) | |
|---|---|---|---|---|---|
| | | Median | Range | Median | Range |
| Control | – | 75 | (2–200) | 200 | (140–390) |
| HuIFN-α (N1) ($2\times10^5$ units) | day 0 | 1[a] | (2–30) | 140[b] | (120–160) |
| HuIFN-α (N1) ($2\times10^5$ units) | day 3 | 13[a] | (0–38) | 205 | (120–230) |
| HuIFN-α (N1) ($2\times10^5$ units) | day 7 | 31[a] | (0–100) | 190 | (150–470) |
| HuIFN-α (N1) ($2\times10^5$ units) | day 14 | 68 | (38–78) | 190 | (140–480) |
| HuIFN-γ ($2\times10^5$ units) | day 0 | 6[a] | (0–40) | 145[c] | (130–500) |

$5\times10^5$ DX3-azac cells were injected i.v. on day 0 and treatment with HuIFN-α (N1) s.c. or rHuIFN-γ i.p. started with 2 h of tumour cell injection. Mice were killed after 5 weeks treatment. 12–14 mice were used per group. Each experiment was performed 4 times. [a]The number of lung nodules was significantly different from control mice (*P*=0.004); [b]The lung weights of mice treated with HuIFN-α (N1) were significantly different from control mice (*P*=0.06); [c]The lung weights of mice treated with rHuIFN-γ were significantly different from control mice (*P*=0.05).

**Figure 1** Percentage survival of mice injected with DX3-azac cells: (--○--) mice treated with 0.2 ml BSA/PBSA (3 mg ml$^{-1}$); (——●——) mice treated with $2\times10^5$ units HuIFN-α (N1); (——■——) mice treated with $2\times10^5$ units rHuIFN-γ. Therapy was initiated 2 h after tumour cell injection and continued daily for 5 weeks. 12–14 mice were used per group. Each experiment was repeated 4 times.

**Figure 2** Clearance rates of $^{125}$Iodo-deoxyuridine-labelled DX3-azac cells: (--○--) mice treated with 0.2 ml BSA/PBSA (3 mg ml$^{-1}$); (——●——) mice treated with $2\times10^5$ units HuIFN-α (N1); (——■——) mice treated with $2\times10^5$ units rHuIFN-γ. Each point represents a mean cpm in lungs of 5 individual animals.

F4/80 positive and TIB 120 positive cells with morphology characteristic of macrophages, occasional Thy 1-2 positive (presumably NK cells) and a few TIB 120 lymphoid cells were present in tumour nodules in both the groups. A semiquantitative estimate of the cells infiltrating the tumours showed no characteristic differences in numbers or distribution between mononuclear cells in control and IFN treated groups.

### *Effect of the clearance of radiolabelled cells*

*In vivo* clearance of radiolabelled DX3-azac cells was measured in control mice and mice treated with HuIFNs. Figure 2 represents the data from one of 3 similar experiments. As shown, all i.v. injected cpm were localised to the lungs by 10 min. By 2 h, 82% of the total injected cpm remained in the lungs and thereafter, the retention of radiolabelled cells was identical in control mice and mice treated with HuIFN-α (N1) or rHuIFN-γ. This suggested that HuIFNs had no effect on the nude mouse organ associated effector cells *in vivo*. By 72 h less than 1% of the total injected cpm survived in the lungs of control mice and mice treated with either IFN.

### *Effect of HuIFNs on nude mouse NK cell activity*

Host lung NK cell activity was measured after treatment with $2\times10^5$ units HuIFN-α (N1) s.c. or $2\times10^5$ units rHuIFN-γ i.p. given daily for 4 days after i.v. injection of DX3-azac cells. The data presented in Table II is representative of 3 typical experiments. As shown, the doses of HuIFNs used in this study had no effect on the nude mouse lung NK cell activity (% lung cytotoxicity at effector target ratio of 100:1, mean±s.d., control=28±1, HuIFN-α (N1)=27±0.8, rHuIFN-γ=28±0.9). Stimulation of NK cell activity was seen when mice were treated with rHuIFN-αA/D a hybrid IFN with equal activity on human and mouse cells (Rehberg *et al.*, 1982).

**Table II** Effect of HuIFN-α (N1) or HuIFN-γ on lung macrophage luna NK cytotoxicity in BALB/c nude mice

| | *% Cytotoxicity ± s.d. Effector:target cell ratio* | | | |
|---|---|---|---|---|
| | *Macrophage* | | *NK* | |
| | *20:1* | *10:1* | *100.1* | *40.1* |
| Control | 15.4±0.9 | 8.3±0.2 | 27.8±1 | 18.4±1 |
| HuIFN-α (N1) (2×10^5 units) | 13.2±1 | 5.4±0.4 | 26.9±0.8 | 17.9±1 |
| rHuIFN-γ (2×10^5 units) | 14 ±0.6 | 7.1±0.5 | 28.0±0.9 | 18.1±4 |
| rHuIFN-α A/D[a] (10^5 units) | N.D. | N.D. | 38.1±1 | 26 ±0.4 |

Mice were treated with daily HuIFN-α (N1) s.c. or rHuIFN-γ i.p. daily for 4 days. Control mice were injected with 0.2 ml PBSA/BSA s.c. daily for 4 days. Four mice were used per group. Each point represents the mean results of 3 groups of mice. COLON 26 were used as targets. [a]rHuIFN-α A/D has equal activity on murine and human cells (Rehberg *et al.*, 1982).

### *Effect of the lung macrophage activity of nude mice*

The effect of HuIFNs on the lung macrophage cytotoxicity was also measured after 4 days. Table II demonstrates the data from one of 3 similar experiments. There was no difference in the lung cytotoxicity between control mice and mice treated with HuIFN-α (N1) or rHuIFN-γ (% macrophage cytotoxicity at effector target ratio 20:1, control = 15±0.9, HuIFN-α (N1) = 13.2±1, HuIFN-γ = 14±0.6).

### *Effect of HuIFN therapy on nude mouse tissue 2-5A synthetase levels*

The 2-5A synthetase activity was measured as another marker of the biological effect of HuIFN-α (N1) or rHuIFN-γ on host cells. 2-5A synthetase levels in the spleens and the lungs were assayed after 4 days of daily treatment with $2 \times 10^5$ units HuIFN-α (N1) s.c., or $2 \times 10^5$ units rHuIFN-γ i.p. Mice treated s.c. with $2 \times 10^5$ units MuIFN α/β were used as positive controls. As shown in Table III there was no increase in the enzyme levels in either the lungs or the spleens after treatment with HuIFN-α (N1). Treatment with rHuIFN-γ resulted in a slight but insignificant increase ($P = 0.1$) in the lungs and spleen levels of the enzyme in comparison to the levels seen in mice treated with MuIFN α/β.

### *Effect of human IFNs on haematological parameters*

There was no statistical difference in the haemoglobin content ($g\,dl^{-1}$, mean±s.d., control = 16.9±1.1, HuIFN-α (N1) = 16.4±1.0, rHuIFN-γ = 16.4±0.9) or white cell counts ($10^6\,ml^{-1}$, mean±s.d., control = 5.75±0.3, HuIFN-α (N1) = 5.4±0.4, rHuIFN-γ = 5.9±0.3) in samples taken by bleeding tail veins after 4 days of daily treatment with either HuIFNs.

**Table III** Levels of 2-5A synthetase in the mouse lung and spleen

| | *Sp. activity (pmol ATP/OD 260$^{-1}$ h$^{-1}$)* | |
|---|---|---|
| | *Lung mean* | *Spleen mean* |
| Control | <1 | 2 |
| MuIFN-α/β (2×10^5 units) | 3.9[a] | 32[b] |
| HuIFN-α (N1) (2×10^5 units) | <1 | 1.97 |
| HuIFN-γ (2×10^5 units) | 2.1[a] | 4.5 |

Mice were treated with HuIFN-α (N1) or MuIFN-α/β s.c. or rHuIFN-γ i.p. in 0.2 ml BSA/PBSA daily for 4 days. Control mice were injected with 0.2 ml BSA/PBSA s.c. for 4 days. 6 h after the last injection the mice were killed and their lungs and spleen taken out for assays. 6–8 mice were used per group. Each assay was performed 4 times on the post-mitochondrial 5–10 fraction. [a]The 2-5A synthetase activity in mice treated with MuIFN-α/β was significantly different from control mice ($P = 0.001$); [b]The 2-5A synthetase activity in mice treated with MuIFN-α/β was significantly different from control mice ($P = 0.09$).

### *Effect of HuIFN-α (N1) or rHuIFN-γ on tumour cell growth* in vitro

Figure 3 shows a typical experiment in which DX3-azac cells growing in Petri dishes as colonies were treated with varying doses of HuIFN-α (N1) or rHuIFN-γ. With maximum concentrations of $10^4$ units $ml^{-1}$, the growth of DX3-azac colonies was completely inhibited after 3 weeks of incubation. When cells were grown at higher cell densities ($5 \times 10^4$ cells/dish) only a small amount of growth inhibition (33%) was seen at the highest doses with each IFN (data not shown).

### Discussion

Therapy with HuIFN-α (N1) or rHuIFN-γ inhibited experimental metastases of a human melanoma DX3-azac in

**Figure 3** The effect of HuIFN-α (N1) (——●——) and rHuIFN-γ (——■——) on colony number. DX3-azac were plated at $10^3$ cells/dish and medium and IFNs were changed twice weekly. Colonies were counted after 3 weeks. Three dishes were used per IFN dilution. Each experiment was performed 3 times.

BALB/c nude mice and significantly increased the life span of tumour bearing mice.

This inhibition appeared to be due to a direct cytostatic effect on the tumour. DX3-azac cells were sensitive to the growth inhibitory effects of IFNs *in vitro* when cells were grown at low density but the human IFNs had no effect on the nude mouse as measured by lung and spleen NK activity, lung macrophage activity and haematological parameters. Moreover, the clearance of radiolabelled DX3-azac cells was identical in control or treated mice confirming that these HuIFNs had no effect on organ associated effector cells. HuIFN-α (N1) had no significant effect on the 2-5A synthetase levels in the lungs or spleens but rHuIFN-γ caused a small elevation.

Previous human tumour xenografts/nude mouse studies with human IFNs (Balkwill, 1982, 1985) provided evidence for a direct action of IFNs on subcutaneous tumours. The experimental metastases model discussed here also demonstrated that IFNs can have a direct inhibitory effect on the growth of metastatic cells in the lungs.

IFNs could mediate their antitumour effect by direct cytotasis or cytolysis of tumour cells.

IFNs could exert their antimetastatic effect by inducing the differentiation of the tumour cells. IFNs can cause differentiation of adipocytes and myocytes (Rossi, 1986). However IFNs inhibited melangogenesis in cultured B16 melanoma cells (Fisher *et al.*, 1981). In the DX3-azac experiments carried out in this paper there was no increase in melanin secretion in the tumour nodules of IFN treated mice and no change in melanin production in IFN treated cell cultures.

Classically, IFNs have mediated their antimetastatic effects in immunocompetent mice only if given prophylactically, i.e., before tumour challenge. The inhibition of experimental metastases has been suggested to be due to stimulation of NK cells (Hanna & Burton, 1981; Brunda *et al.*, 1984; Nishimura *et al.*, 1985). We have shown (Ramani *et al.*, 1986) that rHuIFN-α A/D (an IFN that has activity on murine cells) given after tumour challenge inhibited the experimental metastases of COLON 26 in BALB/c mice and Beige nude (T and NK cell deficient) mice. In addition, in the COLON 26 model, an increased clearance of radiolabelled tumour cells from the lungs was seen in IFN treated mice. In contrast to the above studies, HuIFN α and γ had no effect on the clearance of DX3-azac cells from the lungs. The exact mechanism by which HuIFN-α A/D inhibited the experimental metastases of COLON 26 is not understood but probably NK cells are not involved. Evidence suggests that an early host-mediated mechanism may play a role in IFNs antimetastatic effect in the COLON 26 model.

IFNs have therefore inhibited metastasis in several different animal models. Metastasis is a complex process involving several steps. It is possible that IFNs being pleiotropic molecules influence several stages.

We conclude that in this model system IFNs have a direct cytostatic effect on tumour cells, and that micrometastases are sensitive to growth inhibitory effects of IFNs *in vivo*.

The authors wish to thank Dr Ian Hart for useful discussions, Dr Jane Ormerod for the DX3-azac cells; and Elaine Moodie for technical assistance with the animals.

### References

BALKWILL, F.R., MOODIE, E.M., FREEDMAN, V. & FANTES, K.H. (1982). Human interferons inhibit the growth of established human breast tumours in the nude mouse. *Int. J. Cancer*, **30**: 231.

BALKWILL, F.R., GOLDSTEIN, L. & STEBBING, N. (1985). Differential action of six human interferons against two human carcinomas growing in nude mice. *Int. J. Cancer*, **35**, 613.

BALKWILL, F.R. & PROIETTI, E. (1986). The effects of mouse interferons on human interferons against two human carcinomas growing in nude mice. *Int. J. Cancer*, **38**, 375.

BROUTY-BOYE, D. & GRESSER, I. (1981). Reversibility of the transformed and neoplastic phenotype. *Int. J. Cancer*, **28**, 165.

BRUNDA, M.J., ROSENBAUM, D. & STERN, L. (1984). Inhibition of experimentally induced metastases by recombinant alpha interferon: Correlation between the modulatory effect of interferon treatment on natural killer activity and inhibition of metastases. *Int. J. Cancer*, **34**, 421.

DOUGHERTY, G.J., ALLEN, C.A. & HOGG, N.M. (1986). Applications of immunoperoxidase techniques to the study of tumour host relationships. In *Applications of Immunological Methods in Biological Sciences*, Weir, D.M. *et al.* (eds) p. 125.

FISHER, P.B., MUFSON, R.A. & WEINSTEIN, B. (1981). Interferon inhibits melanogenesis in B-16 mouse melanoma cells. *Biol. Biophys. Res. Comms.*, **100**, 823.

FRIEDMAN, R. & VOGEL, S. (1983). Interferons – special emphasis on the immune system. *Advanc. Immunol.*, **34**, 96.

GLASGOW, L.A. & KERN, E.R. (1981). Effect of interferon administration on pulmonary oesteogenic sarcoma in an experimental murine model. *J. Natl Cancer Inst.*, **67**, 207.

GRESSER, I. (1985). How does interferon inhibit tumour growth? In *Interferon 6*, Gresser I. (ed) p. 93. Academic Press: London.

GRESSER, I. & BOURALI-MAURY, G. (1972). Inhibition by interferon preparations of a solid malignant tumor and pulmonary metastases in mice. *Nature* (New Biol.), **236**, 78.

GRESSER, I. & TOVEY, M.G. (1978). Antitumour effects of interferon. *Biochem. Biophysica Acta*, **516**, 231.

HANNA, N. & BURTON, R.C. (1981). Definite evidence that natural killer (NK) cells inhibit experimental tumor metastasis *in vivo*. *J. Immunol.*, **127**: 1754.

HERBERMAN, R.B., NUNN, M.E. & HOLDEN, H.T. (1978). Low density of Thyl antigen on mouse effector cells mediating natural cytotoxicity against tumour cells. *J. Immunol.*, **121**, 302.

HSU, A., RAINE, L. & FANGER, H. (1981). Use of Avidin-Biotin-Peroxidase conjugates (ABC). *Immuno-peroxidase Techniques*, **29**, 577.

HUME, D.A., ROBINSON, A.P., MACPHERSON, G.G. & GORDON, S. (1983). The mononuclear phagocyte system of mouse defined by immunohistochemical localisation of antigen F4/80. *J. Exp. Med.*, **158**, 1522.

JONAK, G.J. & KNIGHT, E.J. (1986). Interferons and regulation of oncogenes. In *Interferon 7*, Gresser, I. (ed) p. 167. Academic Press: London.

NISHIMURA, J., MITSUI, K., ISHIKAWA, T. & 4 others (1985). Antitumour and antimetastatic activities of human recombinant interferon alpha A/D. *Clin. Exp. Metastasis*, **3**, 295.

ORMEROD, E.J., EVERETT, C.A. & HART, I.R. (1986). Enhanced metastatic capacity of a human tumour line following treatment with 5 azacytidine. *Cancer Res.*, **46**: 884.

RAMANI, P., BALKWILL, F.R. & HART, I. (1986). The effect of interferon on experimental metastases in immunocompetent and immunodeficient mice. *Int. J. Cancer*, **37**, 563.

REHBERG, E., KELDER, B., HOAL, E.G. & PETSKA, S. (1982). Specific molecular activities of recombinant and hybrid leucocyte interferons. *J. Biol. Chem.*, **257**, 11497.

ROSA, F.M., COCHET, M.M. & FELLOUS, M. (1986). Interferon and major histocompatibility complex genes: A model to analyse Eukaryotic gene regulation. In *Interferon 7*, Gresser, I. (ed) p. 48. Academic Press: London.

ROSSI, G.B. (1985). Interferons and cell differentiation. In *Interferon 6*, Gresser, I. (ed) p. 31. Academic Press, London.

SILVERMAN, R.M., SKEHEL, J.J., JAMES, T.C., WRESCHNER, D.H. & KERR, I.M. (1983). rRNA cleavage as an index of ppp(A2′p)nA activity in interferon-treated encephalomyocardities virus-injected cells. *J. Virology*, **6**, 1051.

SPIEGEL, R.J. (1987). Clinical overview of Alpha Interferon. *Cancer*, **59**, 526.

SPRINGER, J., BHATTACHARYA, A. & DORF, M.E. (1981). A shared allogenic determinant on Ia antigens encoded by the I-A and I-E sub-regions: evidence for I gene duplication. *J. Immunol.*, **127**, 2488.

TAYLOR-PAPADIMITROU, J. (1980). Effects of interferon on cell growth and function. In *Interferon 2*, Gresser, I. (ed) p. 13. Academic Press: London.